# Genome-scale analysis identifies NEK2, DLGAP5 and ECT2 as promising diagnostic and prognostic biomarkers in human lung cancer

**DOI:** 10.1038/s41598-017-08615-5

**Published:** 2017-08-14

**Authors:** Yuan-Xiang Shi, Ji-Ye Yin, Yao Shen, Wei Zhang, Hong-Hao Zhou, Zhao-Qian Liu

**Affiliations:** 1 Department of Clinical Pharmacology, Xiangya Hospital, Central South University, Changsha 410008; P. R. China;Institute of Clinical Pharmacology, Central South University, Hunan Key Laboratory of Pharmacogenetics, Changsha, 410078 P.R. China; 2 Hunan Province Cooperation Innovation Center for Molecular Taarget New Drug Study, Hengyang, 421001 P.R. China

## Abstract

This study aims to identify promising biomarkers for the early detection of lung cancer and evaluate the prognosis of lung cancer patients. Genome-wide mRNA expression data obtained from the Gene Expression Omnibus (GSE19188, GSE18842 and GSE40791), including 231 primary tumor samples and 210 normal samples, were used to discover differentially expressed genes (DEGs). NEK2, DLGAP5 and ECT2 were found to be highly expressed in tumor samples. These results were experimentally confirmed by quantitative reverse transcription-polymerase chain reaction (qRT-PCR). The elevated expression of the three candidate genes was also validated using the Cancer Genome Atlas (TCGA) datasets, which consist of 349 tumor and 58 normal tissues. Furthermore, we performed receiver operating characteristics (ROC) analysis to assess the diagnostic value of these lung cancer biomarkers, and the results suggested that NEK2, DLGAP5 and ECT2 expression levels could robustly distinguish lung cancer patients from normal subjects. Finally, Kaplan-Meier analysis revealed that elevated NEK2, DLGAP5 and ECT2 expression was negatively correlated with both overall survival (OS) and relapse-free survival (RFS). Taken together, these findings indicate that these three genes might be used as promising biomarkers for the early detection of lung cancer, as well as predicting the prognosis of lung cancer patients.

## Introduction

Lung cancer is one of the leading causes of cancer-related death in the world^1^. Non-small cell lung cancer and small cell lung cancer are two major pathological types of lung cancer. Unfortunately, many patients are diagnosed with advanced lung cancer due to the asymptomatic nature of the early stages and a lack of effective screening modalities, resulting in a very low 5-year survival rate. Despite the development of multimodal treatment strategies in past decades, including surgical resection, chemotherapy, and radiation therapy, the outcomes of lung cancer patients remain unsatisfactory^2^. Therefore, novel biomarkers for diagnosis, prognosis, and drug response are urgently needed.

Gene expression profiles have been shown to provide diagnostic or prognostic information in a variety of cancers^3–6^. Yang *et al*.^7^ demonstrated that MARCKS contributed to constitutive CAF activation in ovarian cancer, and MARCKS overexpression defined a poor prognosis in ovarian cancer patients. Sun *et al*.^8^ investigated the prognostic potential of lncRNAs in diffuse large-B-cell lymphoma (DLBCL), and identified a potential panel of six-lncRNA signature as a composite biomarker for risk stratification of DLBCL patients at diagnosis. However, efforts to translate gene expression- based analytical methods into the clinical application have been met by several obstacles, including a lack of independent validation or inclusion of clinical variables, as well as overall tumor heterogeneity^9^. To overcome these hurdles, our investigation utilized a large number of patients from multiple studies with diverse patient populations.

In the present study, we identified differentially expressed genes that were common among several expression profiles. We selected the target genes from among the 100 differentially expressed genes based on biology. According to the literature, NIMA-related kinase 2 (NEK2), disc large (drosophila) homolog-associated protein 5 (DLGAP5) and epithelial cell transforming 2 (ECT2) are three specific mitosis-associated genes. In this study, CCNB1, CCNB2, CDKN2A, BUB1, BUB1B and TTK were also involved in cell cycle. Deregulated gene expression of mitosis-related factors, which forces chromosomal segregation during cell division, is frequently observed in cancer. The results of high throughput screening were confirmed by qRT-PCR and further validated in the TCGA datasets. The expression levels of NEK2, DLGAP5 and ECT2 were significantly higher in lung cancer patients than in normal subjects. In addition, we explored and discussed the diagnostic and prognostic value of the three genes in lung cancer. ROC analyses showed that NEK2, DLGAP5 and ECT2 levels could also robustly distinguish lung cancer patients from normal subjects, demonstrating high AUC, specificity and sensitivity values. Elevated expression of NEK2, DLGAP5 and ECT2 were both remarkably associated with reduced survival and increased risk of recurrence. Taken together, our findings revealed that NEK2, DLGAP5 and ECT2 might be used as promising biomarkers for the early detection of lung cancer, as well as predicting the prognosis of lung cancer patients.

## Results

### Identification of DEGs between tumor tissues and normal lung tissues

**Figure 1:**
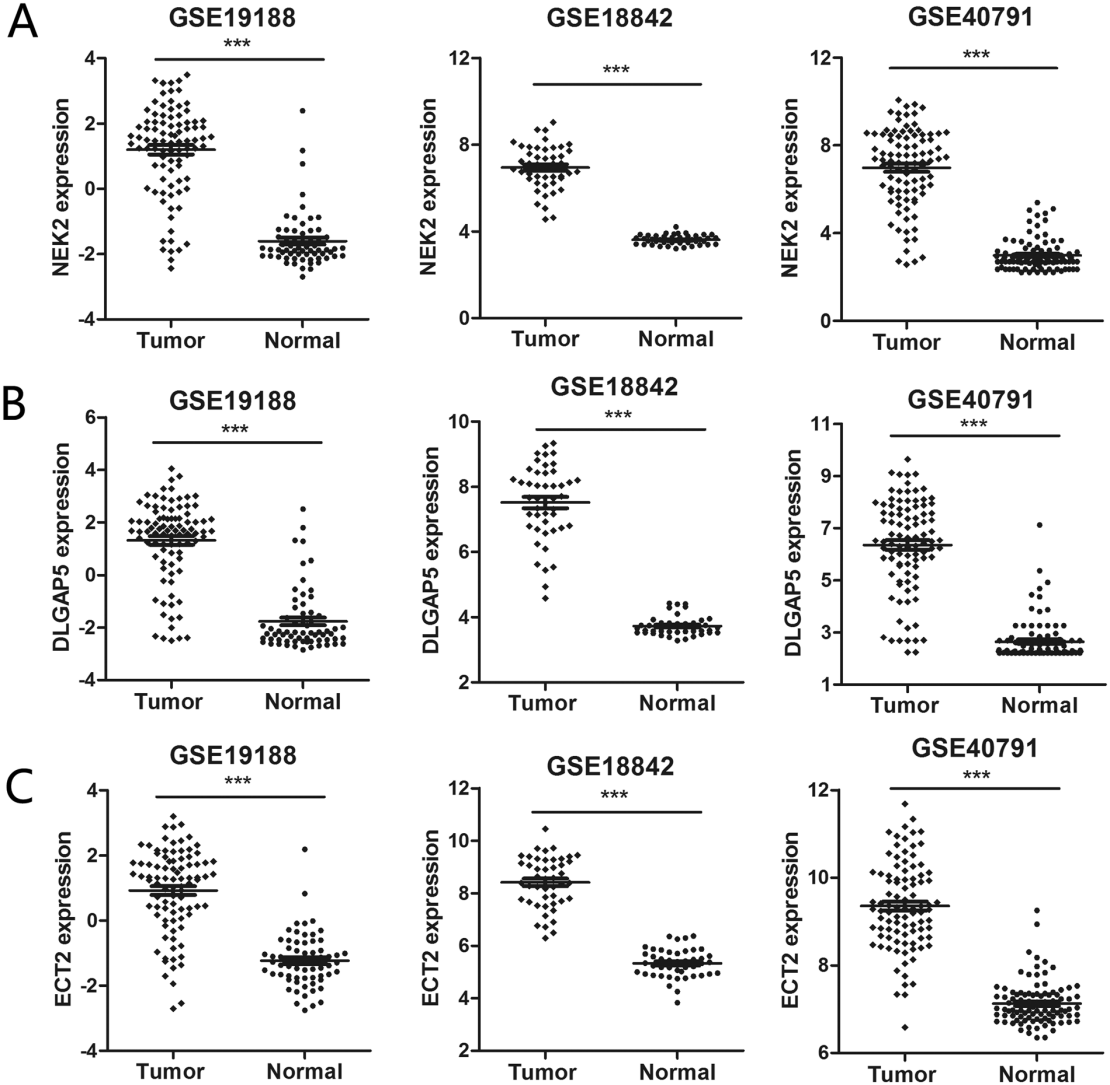
Identification of the differentially expressed genes. (**A**) Identification of mRNA expression of NEK2 in three datasets, respectively. (**B**) Identification of mRNA expression of DLGAP5 in three datasets, respectively. (**C**) Identification of mRNA expression of ECT2 in three datasets, respectively. ***corresponds to P < 0.001; **P < 0.01 and *P < 0.05.

In our study, three expression profiles (GSE19188, GSE18842, GSE40791) were used to identify DEGs between tumors and normal lung tissues. Genes with corrected P-values <0.05 and absolute fold changes >4 were considered as DEGs. The results showed that 131 genes were up-regulated in GSE19188, 316 genes were up-regulated in GSE18842, and 309 genes were up-regulated in GSE40791 (Figure S1A–C). Then, we performed an overlap analysis of the DEGs, a total of 100 genes were significantly up-regulated in the three lung cancer datasets (Figure S1D, Table S2). The increased expression of NEK2, DLGAP5 and ECT2 in lung cancer was identified in three GEO datasets. An unpaired t-test was applied to comparisons of the two groups (tumor vs normal), and p-values of less than 0.05 were considered to be statistically significant (Fig. 1A–C). Importantly, these three genes play an important role in mitosis. Thus, in this study, we focused on NEK2, DLGAP5 and ECT2, three critical mitotic genes.

### Independent validation

**Figure 2:**
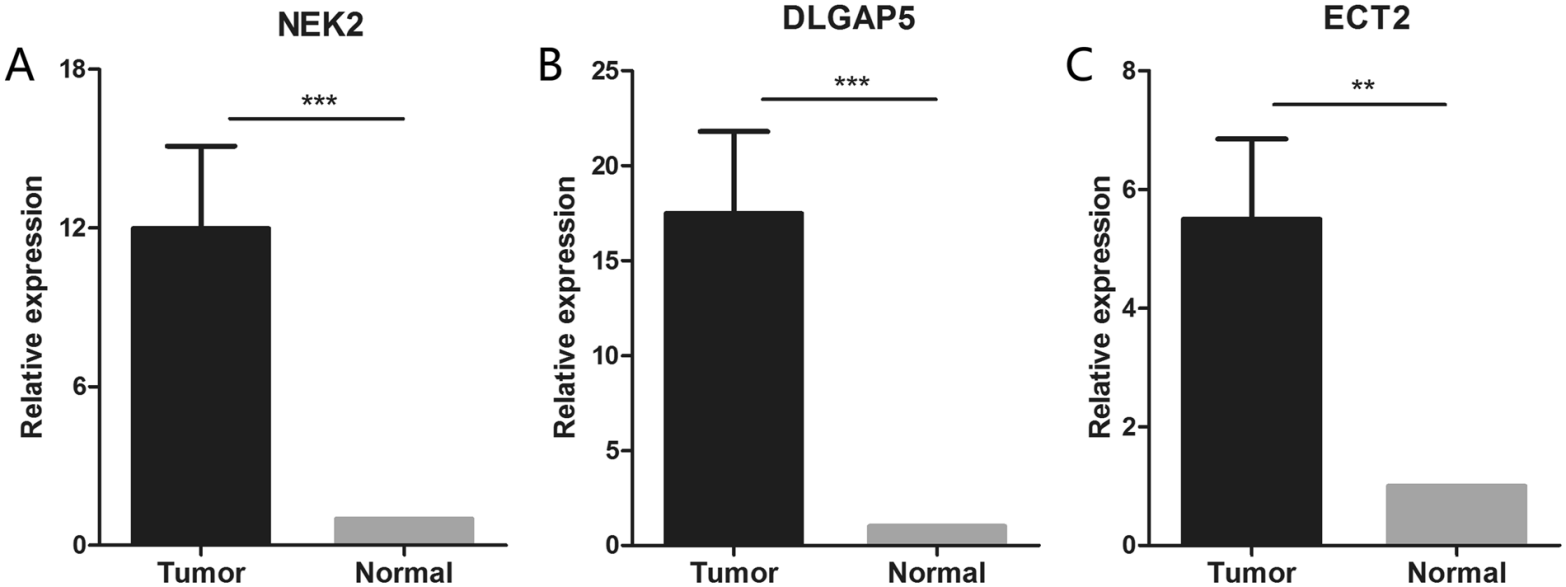
Clinical validation of the selected genes in paired tumor and normal tissues using qRT-PCR. (**A**) NEK2 (**B**) DLGAP5 (**C**) ECT2 ***corresponds to P < 0.001; **P < 0.01 and *P < 0.05.

**Figure 3:**
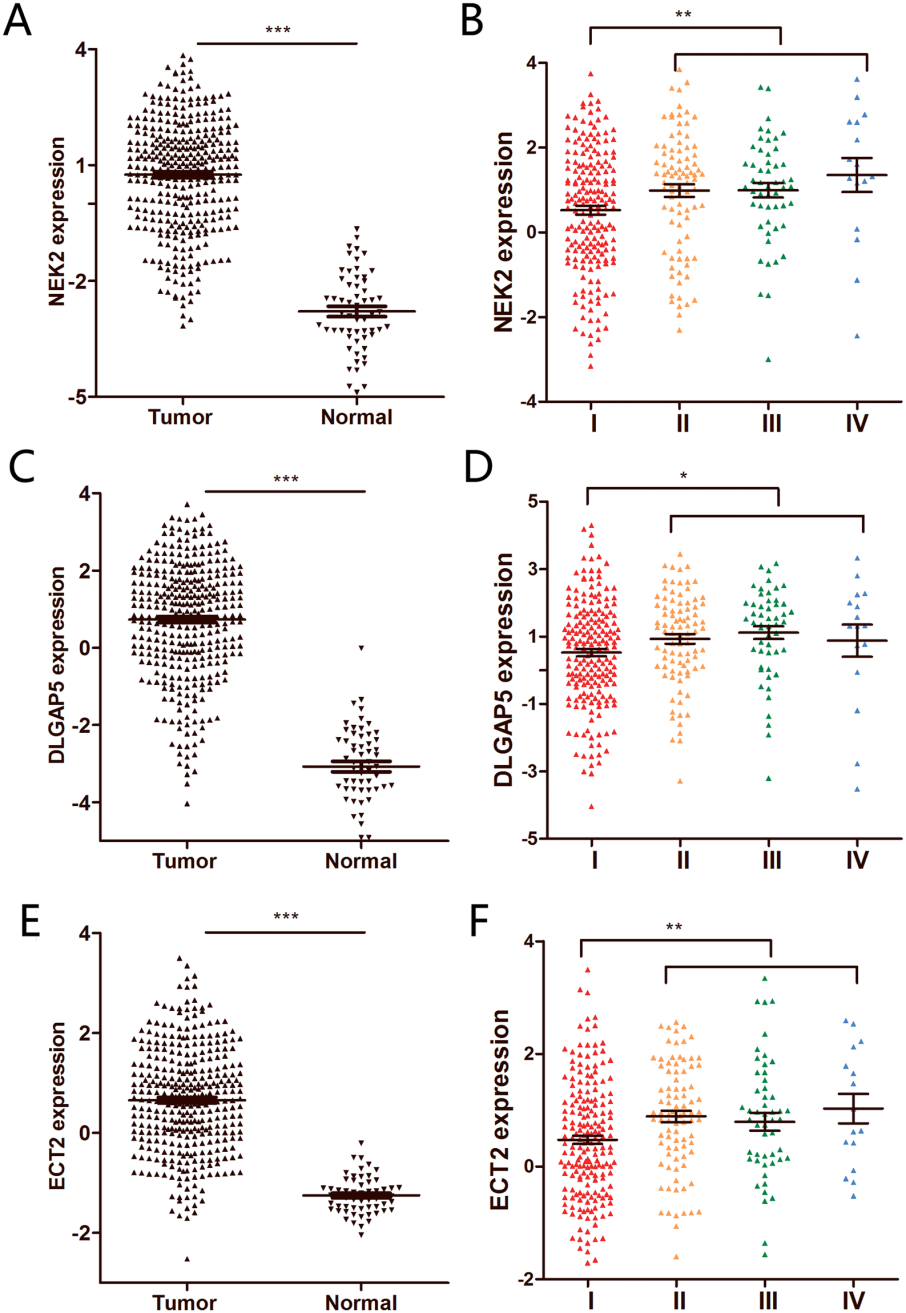
Validation of the selected genes using 349 lung cancer and 58 normal tissues from TCGA datasets. (**A**) Validation of mRNA expression of NEK2 in TCGA datasets. (**B**) Gene expression of NEK2 in lung cancer patients according to clinical stage. **(C**) Validation of mRNA expression of DLGAP5 in TCGA datasets. (**D**) Gene expression of DLGAP5 in lung cancer patients according to clinical stage. **(E**) Validation of mRNA expression of ECT2 in TCGA datasets. (**F**) Gene expression of ECT2zE in lung cancer patients according to clinical stage. ***Corresponds to P < 0.001; **P < 0.01 and *P < 0.05.

To confirm our previous results, we selected a series of DEGs for further investigation using another independent set of 56 paired tumors and normal lung tissues. The clinical characteristics of this cohort are summarized in Table 1. NEK2, DLGAP5 and ECT2 expression levels were significantly elevated in tumor tissues compared with normal lung tissues (Fig. 2A–C). As our study was limited to a small number of patients, we expanded the sample size for further validation by using TCGA datasets. A total of 349 lung cancer and 58 normal tissue samples were selected. The expression levels of NEK2, DLGAP5 and ECT2 were similar to those in our training cohort, with significant differences in expression between tumor and normal (Fig. 3A,C,E), suggesting that the differential expression statuses of these three genes is a common feature of lung cancer. Moreover, the increases in NEK2, DLGAP5 and ECT2 expression levels were clearly discernible between TNM stages, with significantly higher levels in stage II-IV patients compared with stage I patients. (Fig. 3B,D,F).

**Table 1:** Clinicopathological characteristics of patients for clinical validation cohorts.

Clinical and pathological variables	Clinical validation cohorts (N = 56)
Age (years)	
<60	29
≥60	27
Gender	
Male	54
Female	2
Smoking status	
Smoker	48
Non-smoker	8
Clinical stage	
I-II	28
III-IV	28
Differentiation	
Well	8
Moderate	27
Poor	21
Lymph node metastasis	
Yes	21
No	35

### Correlation between the three biomarkers and clinicopathologic variables

Next, the analysis of the associations between DEG expression and clinicopathological characteristics are presented in Table 2. The TCGA dataset was used for correlation analyses. NEK2 expression was significantly associated with age (P = 0.027), gender (P < 0.001), clinical stage (P = 0.033), pathologic T stage (P < 0.001) and therapy outcome (P = 0.004). Elevated DLGAP5 expression was significantly correlated with all six clinicopathologic variables. No significant association was observed between ECT2 expression and patient age or clinical stage. Table 2 shows the significant associations between high ECT2 expression in lung cancer and gender (P = 0.002), new tumor event (P = 0.026), pathologic T stage (P = 0.002), and therapeutic outcome (P = 0.012). These results suggest that expression changes in NEK2, DLGAP5 and ECT2 may play a vital role in lung cancer progression.

**Table 2:** Correlation between NEK2/ DLGAP5/ECT2 expression and clinical characteristics in 349 lung cancer patients.

Characteristic	n = 349	NEK2 expression levels			DLGAP5 expression levels			ECT2 expression levels		
		low (n = 174)	high (n = 175)	P	low (n = 174)	high (n = 175)	P	low (n = 174)	high (n = 175)	P
**Age (years)**										
<65	159	69	90	**0.027**	69	90	**0.027**	74	85	0.257
≥65	190	105	85		105	85		100	90	
**Gender**										
Female	190	113	77	**<0.001**	114	76	**<0.001**	109	81	**0.002**
Male	159	61	98		60	99		65	94	
**Clinical stage**										
I & II	281	148	133	**0.033**	149	132	**0.016**	144	137	0.292
III & IV	68	26	42		25	43		30	38	
**New tumor event**										
YES	99	40	59	0.26	39	60	**0.014**	40	59	**0.026**
NO	250	134	116		135	115		134	116	
**Pathologic T stage**										
T1	127	82	45	**<0.001**	79	48	**0.001**	79	48	**0.002**
T2	180	76	104		74	106		77	103	
T3 + T4	40	16	24		21	21		18	24	
**Therapy outcome**										
CR + PR	145	81	64	**0.004**	81	64	**0.01**	82	63	**0.012**
SD + PD	86	31	55		33	53		34	52	

CR: Complete response; PR: Partial response; SD: Stable disease; PD: Progressive disease. Response Evaluation Criteria In Solid Tumors (RECIST) has a detailed definition.

### Diagnostic value of NEK2, DLGAP5 and ECT2 in lung cancer

**Figure 4:**
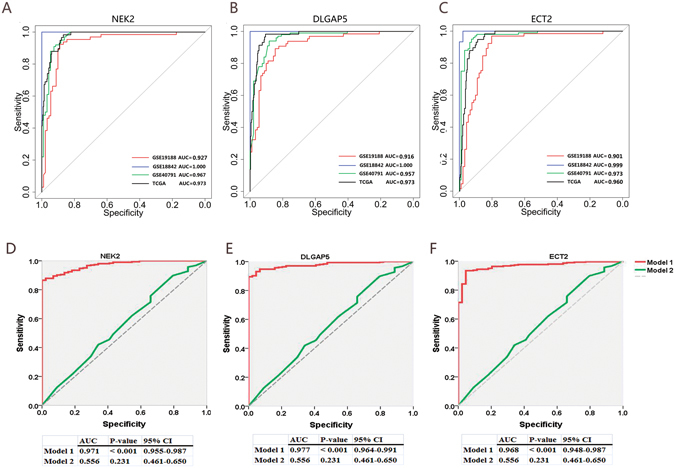
Diagnostic value of the three candidate genes in lung cancer by ROC curves analysis. (**A**) NEK2 (**B**) DLGAP5 (**C**) ECT2. The four datasets are marked in the figures. The red line is GSE19188, the blue line is GSE18842, the green line is GSE40791, and the black line is TCGA datasets. For the ROC curve, the comparisions between model with the target gene (Model 1) and the model without the target gene (Model 2) was performed. The Model 1 (red line) includes age, gender, smoking status, clinical stage and the target gene. The Model 2 (green line) without the target gene. **(D)** NEK2. AUC_Model 1_ = 0.971 (P-value <0.001), AUC _Model 2_ = 0.556 (P-value = 0.231). **(E)** DLGAP5. AUC_Model 1_ = 0.977 (P-value <0.001), AUC _Model 2_ = 0.556 (P-value = 0.231). **(F)** ECT2. AUC_Model 1_ = 0.968 (P-value <0.001), AUC _Model 2_ = 0.556 (P-value = 0.231).

Subsequently, ROC analysis was performed to assess the diagnostic value of NEK2, DLGAP5 and ECT2 as biomarkers detecting lung cancer. The AUC of tumor and normal groups in NEK2 analyses were significantly different for all four lung cancer datasets, with the following values: AUC_GSE19188_ = 0.927 (sensitivity: 0.923, specificity: 0.890), AUC_GSE18842_ = 1 (sensitivity: 1, specificity: 1), AUC_GSE40791_ = 0.967 (sensitivity: 0.910, specificity: 0.926) and AUC _TCGA_ = 0.977 (sensitivity: 0.983, specificity: 0.873) (Fig. 4A, Table 3). Similarly, ROC analyses showed that DLGAP5 and ECT2 levels could also robustly distinguish lung cancer patients from normal subjects, demonstrating high AUC, specificity and sensitivity values (Fig. 4B–C, Table 3). Furthermore, in order to exclude the influence of primary clinical factors (age, gender, clinical stage, smoking history) on target gene performance, we further constructed prediction models including (Model 1) or excluding (Model 2) the target gene. Model 1 includes clinical factors and the target gene. Model 2 includes only clinical factors, and excludes the target gene. We compared these models, and the results of these comparisons are shown in Table S3 and Fig. 4D–F. Model 2 performed worse than Model 1. These results suggest that these target genes are important factors for maintaining the model’s performance. Collectively, our results suggest that NEK2, DLGAP5 and ECT2 could be suitable biomarkers for lung cancer diagnosis.

**Table 3:** ROC curve analyses using NEK2/DLGAP5/ECT2 for distinguishing patients with lung cancer from normal control subjects.

	Lung cancer datasets	AUC value	P value	95% CI	Cut off value	Specificity, sensitivity
**NEK2**	GSE19188	0.9272	<0.0001	0.8826–0.9719	−0.718	0.890, 0.923
	GSE18842	1	<0.0001	1.0000–1.0000	4.384	1.000, 1.000
	GSE40791	0.9665	<0.0001	0.9415–0.9915	3.991	0.926, 0.910
	TCGA	0.9733	<0.0001	0.9647–0.9883	−0.871	0.868, 0.983
**DLGAP5**	GSE19188	0.9158	<0.0001	0.8703–0.9614	−0.401	0.846, 0.892
	GSE18842	1	<0.0001	1.0000–1.0000	4.505	1.000, 1.000
	GSE40791	0.9572	<0.0001	0.9297–0.9847	4.043	0.883, 0.940
	TCGA	0.973	<0.0001	0.9578–0.9881	−1.338	0.905, 0.983
**ECT2**	GSE19188	0.9013	<0.0001	0.8495–0.9530	−0.008	0.802, 0.969
	GSE18842	0.9986	<0.0001	0.9950–1.002	6.439	0.978, 1.000
	GSE40791	0.9728	<0.0001	0.9488–0.9967	8.008	0.926, 0.950
	TCGA	0.9601	<0.0001	0.9421–0.9782	−0.628	0.888, 0.948

### Prognostic value of NEK2, DLGAP5 and ECT2 in lung cancer

Furthermore, in order to assess the prognostic value of NEK2, DLGAP5 and ECT2 as biomarkers for lung cancer, we investigated the association between the expression levels of each of these targets with survival through Kaplan-Meier analysis. We used the log-rank test in 349 lung cancer patients. The Cox proportional hazards regression model was also used to evaluate the predictive value of NEK2, DLGAP5 and ECT2 mRNA levels in lung cancer patients. Two types of survival outcomes were considered in survival analyses. Overall survival (OS) was defined as the time between the date of surgery and date of death or last follow-up, and relapse-free survival (RFS) was defined as the period from surgery to recurrence or last follow-up.

**Figure 5:**
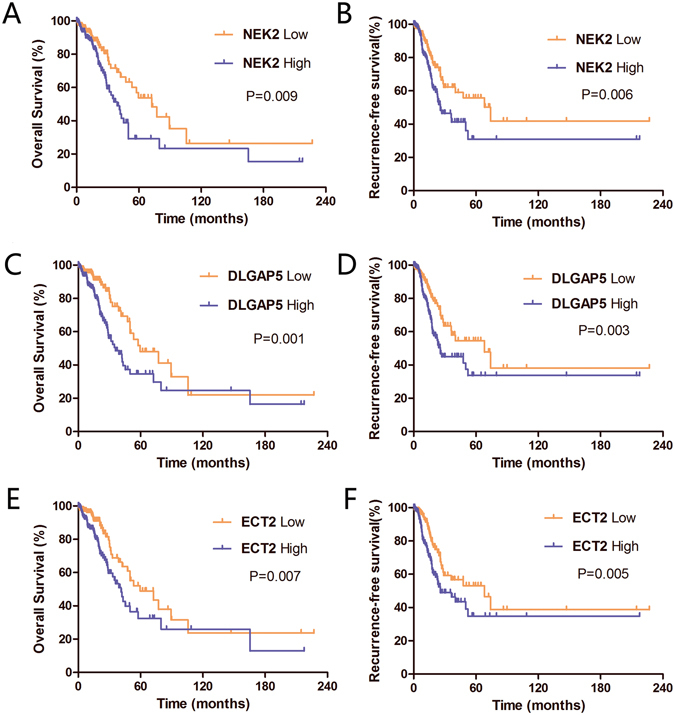
Kaplan- Meier analysis of OS and RFS probabilities based on the expression levels of three candidate genes. (**A**,**C**,**E**) Survival curves of lung cancer patients according to the status of NEK2/DLGAP5/ECT2 expression levels. Patients with high NEK2/DLGAP5/ECT2 expression showed significantly poorer OS than those with low NEK2/DLGAP5/ECT2 expression (P = 0.009, P = 0.001; P = 0.007, respectively). **(B**,**D**,**F**) RFS of lung cancer patients according to the status of NEK2/DLGAP5/ECT2 expression levels. Patients with high NEK2/DLGAP5/ECT2 expression showed significantly poorer RFS than those with low NEK2/DLGAP5/ECT2 expression (P = 0.006, P = 0.003; P = 0.005, respectively).

In this study, the TCGA dataset was used for prognostic analyses. We divided expression levels into two categories using the median. High expression levels were classified as those that were above the median, while low expression levels were below the median. On the whole, patients with low NEK2 levels had statistically longer OS (P = 0.009; Fig. 5A) and RFS (P = 0.006; Fig. 5B) than those with high NEK2 levels. The median OS in NEK2 low expression group is 72.5 months, in NEK2 high expression group is 39 months. The median RFS in NEK2 low expression group is 73.9 months, in NEK2 high expression group is 25.7 months. Similarly, DLGAP5 expression was significantly related with OS (P = 0.001; Fig. 5C) and RFS (P = 0.003; Fig. 5D) of lung cancer patients. The median OS in the low and high DLGAP5 expression groups is 59.7 months and 35.8 months, respectively. The median RFS in the low and high DLGAP5 expression groups is 68.2 months and 25.7 months, respectively. These figures revealed that higher DLGAP5 expression correlated with a worse prognosis and earlier recurrence. Elevated expression of ECT2 was also remarkably associated with reduced survival (P = 0.007; Fig. 5E) and increased risk of recurrence (P = 0.005; Fig. 5F). The median OS in low and high ECT2 expression groups is 59.7 months and 41.2 months, respectively. The median RFS in low and high ECT2 expression groups is 68.2 months and 25.7 months, respectively. Taken together, high expression of these three genes were all remarkably associated with reduced survival and increased risk of recurrence. The univariate/multivariate analyses were carried out to evaluate the target genes and other factors using a Cox proportional hazard regression model. The results showed that the expression of each target gene was significantly correlated with the prognosis of lung cancer patients (Table 4).

**Table 4:** Univariate and multivariate Cox regression analyses for overall survival and recurrence-free survival.

Variable	OS		RFS	
	HR (95% CI)	P-value	HR (95% CI)	P-value
**Univariate analysis**				
Age (<65 vs. ≥65)	1.262(0.826–1.929)	0.281	1.415(0.943–2.123)	0.094
Gender (Female vs. Male)	1.172(0.774–1.775)	0.454	0.992(0.666–1.477)	0.968
Clinical stage (I–II vs. III–IV)	2.439(1.579–3.768)	**<0.001**	1.453(0.911–2.318)	0.117
Smoking history (yes vs. no)	0.815(0.516–1.286)	0.378	0.847(0.551–1.301)	0.448
NEK2 (low vs. high)	1.749(1.140–2.681)	**0.010**	1.738(1.161–2.601)	**0.007**
DLGAP5 (low vs. high)	1.986(1.285–3.070)	**0.002**	1.798(1.201–2.692)	**0.004**
ECT2 (low vs. high)	1.779(1.162–2.722)	**0.008**	1.752(1.172–2.619)	**0.006**
**Multivariate analysis**				
Age (<65 vs. ≥65)	1.719(1.050–2.815)	**0.031**	1.919(1.210–3.043)	**0.006**
Gender (Female vs. Male)	1.218(0.780–1.903)	0.386	0.991(0.650–1.513)	0.968
Clinical stage (I–II vs. III–IV)	2.372(1.484–3.790)	**<0.001**	1.400(0.845–2.318)	0.191
Smoking history (yes vs. no)	0.826(0.495–1.378)	0.464	0.779(0.489–1.241)	0.294
NEK2 (low vs. high)	1.718(1.073–2.751)	**0.024**	1.771(1.142–2.745)	**0.011**
Age (<65 vs. ≥65)	1.880(1.131–3.123)	**0.015**	1.978(1.240–3.153)	**0.004**
Gender (Female vs. Male)	1.155(0.736–1.811)	0.531	0.935(0.610–1.432)	0.756
Clinical stage (I–II vs. III–IV)	2.388(1.493–3.821)	**<0.001**	1.400(0.846–2.319)	0.191
Smoking history (yes vs. no)	0.826(0.492–1.386)	0.469	0.769(0.482–1.226)	0.270
DLGAP5 (low vs. high)	2.101(1.294–3.412)	**0.003**	1.895(1.218–2.949)	**0.005**
Age (<65 vs. ≥65)	1.586(0.971–2.590)	0.066	1.742(1.101–2756)	**0.018**
Gender (Female vs. Male)	1.242(0.793–1.945)	0.344	1.005(0.659–1.533)	0.982
Clinical stage (I–II vs. III–IV)	2.501(1.565–3.996)	**<0.001**	1.465(0.886–2.421)	0.137
Smoking history (yes vs. no)	0.820(0.491–1.372)	0.450	0.770(0483–1.227)	0.272
ECT2 (low vs. high)	1.805(1.142–2.853)	**0.012**	1.699(1.105–2.612)	**0.016**

Further subgroup analysis, stratified by clinicopathological features, were perfomed to explore the effects of NEK2 expression on OS and RFS in the patients. In patient groups characterized as female, age <65, stage T3 + T4, or in groups with new tumor events, there was no difference in OS between NEK2-low and NEK2-high patients. Meanwhile, in groups characterized as age ≥65, male, stage T1 + T2, patients with low NEK2 levels had statistically better OS than those with high NEK2 levels (P = 0.019, Figure S2A; P = 0.011, Figure S2B; P = 0.036, Figure S2C, respectively). Similarly, Kaplan-Meier analysis revealed that groups with high NEK2 levels had poor RFS, which was significantly associated with groups age ≥65 (P = 0.012, Figure S2D), male (P = 0.034, Figure S2E), and stage T1 + T2 (P = 0.004, Figure S2F). In groups characterized as age <65 (or ≥65), male, stage T3 + T4, the patients with low DLGAP5 levels had statistically better OS than those with high DLGAP5 levels (P = 0.035, P = 0.002, Figure S3A; P = 0.020, Figure S3B; P = 0.021, Figure S3C, respectively). Our results also showed that groups with high DLGAP5 levels had poor RFS, which was significantly associated with groups age ≥65 (P = 0.009, Figure S3D), female (P = 0.006, Figure S3E), and stage T1 + T2 (P = 0.038, Figure S3F). Kaplan-Meier analysis revealed that groups with low ECT2 levels had better OS, which was significantly associated with groups age <65 (P = 0.005, Figure S4A), male (P = 0.004, Figure S4B), and stage T3 + T4 (P = 0.023, Figure S4C). Similarly, low ECT2 levels had a better RFS which significantly associate with age <65 (P = 0.008, Figure S4D), male (P = 0.033, Figure S4E), and stage T1 + T2 (P = 0.041, Figure S4F).

## Discussion

Lung cancer remains the most common cause of cancer related death worldwide^1^. The high mortality among patients with lung cancer is mainly due to the absence of an effective screening strategy to identify lung cancer in early stages^10^. Current screening strategies for lung cancer include conventional radiography, sputum cytology, and more recently, low-dose computed tomography (LDCT). LDCT screening can significantly improve early diagnosis and reduce lung cancer mortality. However, the false-positive rate is high for screening with LDCT and this can lead to harm due to unnecessary workups of benign nodules^11, 12^. For many decades, cytotoxic chemotherapy was the most effective treatment to improve overall survival and life quality in these patients, despite its many drawbacks^13^. At the same time, researchers made substantial efforts towards the development of molecular targeted agents^14^. Systematic clinical studies and basic research on lung cancer has improved the survival; however, the long-term outcomes of lung cancer patients remain poor. Thus, it is necessary to identify new biomarkers to improve the diagnosis and prognosis of lung cancer.

NEK2 is a serine/threonine kinase that is involved in regulation of centrosome duplication and spindle assembly during mitosis^15, 16^. Dysregulation of these processes causes chromosome instability (CIN) and aneuploidy, which are hallmark changes in many tumors^17, 18^. NEK2 exists in three alternative splice isoforms, which are NEK2A, NEK2B and NEK2C^19^. NEK2 overexpression has been observed in several human cancers. Increased expression of NEK2 has been reported to be involved in tumor progression and is associated with poor prognosis in pancreatic ductal adenocarcinoma^20^, prostate cancer^21^, colon cancer^22^. However, the association between the expression level of NEK2 and the early diagnosis of lung cancer patients remains to be rigorously and systematically evaluated. ECT2 is a BRCT-containing protein whose function has been best studied in cytokinesis. He *et al*.^23^ showed that ECT2 is located to the chromatin and DNA damage foci-like structures and it facilitates PIKK-mediated phosphorylation of p53 on Ser15, the execution of apoptosis, and the activation of S and G2/M checkpoints. Luo *et al*.^24^ showed that elevated expression of ECT2 predicts an unfavorable prognosis in patients with colorectal cancer. Another potential predictor of lung cancer diagnosis and prognosis is DLGAP5. DLGAP5 is a mitotic spindle protein that promotes the formation of tubulin polymers resulting in tubulin sheets around the end of the microtubules^25^. DLGAP5 contains a guanylate-kinase-associated protein (GKAP) domain that is conserved among various species. This domain is also found in many eukaryotic signaling proteins, suggesting that DLGAP5 may have important biological functions as a signaling molecule^26^. DLGAP5 is involved in cancer formation and progression, suggesting that the gene and its product may be potential therapeutic targets^27^.

NEK2, DLGAP5 and ECT2 are mitosis-associated genes that play an important role in tumorigenesis. At present, these genes have been reported to be involved in lung cancer development. Through clustering of a genome-scale co-expression network, lung adenocarcinoma modules were revealed; in few modules, the genes such as DLGAP5 and BIRC5 are present that play a crucial role in cell cycle progression^28^. Das *et al*.^29^ uncovered a novel role for Nek2 in promoting tumorigenesis by regulating an axis of metastasis and cell survival. Ect2 regulates rRNA synth-esis through a PKCi-Ect2-Rac1-NPM signaling axis that is required for lung tumorigenesis^30^. It is of great clinical significance to explore the early diagnosis and prognosis of these three genes. In previous studies, there are some studies on the association between gene overexpression and poor prognosis in lung cancer. Zhong *et al*.^31^ discovered that the patients with overexpressed NEK2, Mcm7 and Ki67 had a poorer overall survival time compared to those with low expression for all stages. Landi *et al*.^32^ showed that the very mitotic genes (NEK2 and TTK) known to be involved in cancer development are induced by smoking and affect survival. Schneider *et al*.^33^ found that the expression of the mitosis-associated genes AURKA, DLGAP5, TPX2, KIF11 and CKAP5 is associated with the prognosis of NSCLC patients. ECT2 overexpression may be a useful index for application of adjuvant therapy to lung cancer patients who are likely to have poor clinical outcome^34, 35^. However, some genes identified with prognostic implications in one cohort might be difficult to be verified in other cohorts. The high reliability and reproducibility of the microarray technology in identifying the target genes are also essential for its application in discovering the clinical biomarkers.

Microarray technology has substantially enhanced the search for biomarkers for cancer diagnosis and prognosis. In this study, we identified and validated the expression of NEK2, DLGAP5 and ECT2 in multiple lung cancer datasets, and the results showed that the expression levels of these three genes were significantly higher in lung cancer patients than in normal subjects. Importantly, the expression levels of the three candidate genes were significantly associated with clinicopathologic variables. Furthermore, we revealed the diagnostic and prognostic value of the candidate genes. These cancer biomarkers can be used for early detection, disease monitoring and risk assessment. However, there are some limitations in this study. We just examined the expression of the target genes in tissue samples. Because the ultimate goal of biomarker is specific, early and non-invasive diagnosis and post-therapy monitoring of cancer, body fluid (plasma, urine and sputum) has been thought as an appropriate biological material. In the future, we will also detect the expression of these biomarkers in body fluid samples.

Taken together, these findings indicate that NEK2, DLGAP5 and ECT2 overexpression might be used as promising biomarkers for the diagnosis and prognosis of lung cancer. These genes may also serve as potential therapeutic targets in lung cancer. More work is needed to elucidate the function of these three candidate genes and their roles in tumorigenesis.

## Materials and Methods

### Patients and tissue samples

Fifty-six patients from Xiangya Hospital (Changsha, China) were included in this study. All the patients provided written informed consent. Experiments and procedures were performed in accordance with the Helsinki Declaration of 1975; and were approved by the Ethics Committee of Xiangya School of Medicine, Central South University. Tumor and matched distant (>5 cm) normal lung tissue samples were collected from NSCLC patients who underwent resection for primary lung cancer. All fresh tissues were frozen in liquid nitrogen immediately after resection and stored at −80 °C. Their basic clinical characteristics were summarized in Table 1.

### Lung cancer gene expression datasets

Three lung cancer datasets (GSE19188, GSE18842, GSE40791) generated from the Affymetrix platform and corresponding clinical information of lung cancer patients were retrieved from the Gene Expression Omnibus (http://www.ncbi.nlm.nih.gov/geo). GSE19188, including 91 tumors and 65 adjacent normal lung tissues, GSE18842, which includes 46 tumors and 45 controls, and GSE40791 containing 94 tumors and 100 non-tumor tissues.

Validation datasets were acquired from the Cancer Genome Atlas (TCGA) data portal (http://tcga-data.nci. nih.gov). This data set contains 349 adenocarcinomas and 58 non-tumor tissues with both mRNA expression data and clinical feature information available for performing the Receiver Operating Curves (ROC) analysis, survival analysis and correlation analysis. The aim of this study was to identify promising biomarkers for the early detection of lung cancer and to evaluate the prognosis of lung cancer patients. The latest version of the TCGA LUAD dataset includes 571 samples (513 tumors and 58 normal tissues). Two recurrent tumor samples were removed, 28 samples lacking OS data were removed, 133 samples lacking RFS data were removed, and 1 sample lacking clinical stage data was removed, and finally retained the 349 adenocarcinoma samples (primary tumor) and 58 non-tumor samples. Detailed clinical information of patients used in this study was shown in Table 2.

### mRNA expression profiling using microarrays

Raw microarray data files (.CEL files) of the three datasets were analyzed using the Robust Multichip Average (RMA) algorithm by the R package Affy^36^. After that, the Linear Models for Microarray Data (LIMMA) package in R was used to calculate the probability of probes being differentially expressed between cases and controls^37^. P value correction was performed using the Benjamini-Hochberg (BH) FDR from the package in R. Corrected P-values <0.05 and absolute fold changes >4 were used to identify significantly DEGs. All data analysis were performed using R (http://www.r-project.org/, version 2.15.0) and Bioconductor^38^. Visualization of the DEGs including heat map, volcano plot and venn diagram was achieved by using gplots, lattice, and venn diagram packages in R, respectively.

### Quantitative reverse transcription-polymerase chain reaction (qRT-PCR)

Total RNA was extracted from samples with Trizol reagent (Takara, Dalian, China) and then reverse transcribed to cDNA using PrimeScriptTM RT-PCR Kit (Takara, Dalian, China) following the manufacturer’s instructions. Real-time PCR was performed using SYBR® Premix DimerEraser™ (Perfect Real Time) (Takara, Dalian, China) in Roche LightCycler 480 II Real-Time PCR system (Roche Diagnostics Ltd., Rotkreuz, Switzerland). Primers used for real-time PCR are shown in Supplementary Table 1. The threshold cycle value (Ct) of each product was determined and normalized against that of the internal control GAPDH. The differences in mRNA expression levels were compared by t test using SPSS 18.0 (SPSS Inc, Chicago, Illinois, USA). P-values of less than 0.05 were considered statistically significant.

### Statistical analysis

The SPSS version 18.0 (Chicago, IL) and Prism 5.0 GraphPad software (San Diego, CA) were used for statistical analysis. Student’s t-test was applied for comparisons of two groups. ROC curves were used to assess the diagnostic value of each marker^39^. Area under the curve (AUC) was computed for each ROC curve, and 95% confidence intervals (CI) were also estimated by bootstrapping with 1,000 iterations. Survival analysis was carried out according to Kaplan–Meier analysis and the Log-rank test. The Cox proportional hazards regression model was applied to perform univariate and multivariate analyses. P-values of less than 0.05 were considered statistically significant.

## Electronic supplementary material

Supplementary materials

## References

[1] Siegel RL, Miller KD, Jemal A (2015). Cancer statistics, 2015. CA Cancer J Clin.

[2] Zappa C, Mousa SA (2016). Non-small cell lung cancer: current treatment and future advances. Translational Lung. Cancer Research.

[3] Vychytilova-Faltejskova, P. *et al*. Serum-based microRNA signatures in early diagnosis and prognosis prediction of colon cancer. Carcinogenesis, doi:bgw078 10.1093/carcin/bgw078 (2016). 10.1093/carcin/bgw078 27485599

[4] Imaoka, H. *et al*. Circulating microRNA-1290 as a novel diagnostic and prognostic biomarker in human colorectal cancer. Annals of Oncology, mdw 279, doi:10.1093/annonc/mdw279 (2016). 10.1093/annonc/mdw279 27502702

[5] Yang B (2016). MicroRNA-21 in peripheral blood mononuclear cells as a novel biomarker in the diagnosis and prognosis of prostate cancer. Cancer Biomarkers.

[6] Sun L (2016). CPA4 is a Novel Diagnostic and Prognostic Marker for Human Non-Small-Cell Lung Cancer. Journal of Cancer.

[7] Yang, Z. *et al*. MARCKS contributes to stromal cancer-associated fibroblast activation and facilitates ovarian cancer metastasis. Oncotarget, doi:10.18632/oncotarget.8726 (2016). 10.18632/oncotarget.8726 PMC5122339 27081703

[8] Sun J (2016). A potential panel of six-long non-coding RNA signature to improve survival prediction of diffuse large-B-cell lymphoma. Scientific Reports.

[9] Subramanian J, Simon R (2010). Gene Expression-Based Prognostic Signatures in Lung Cancer: Ready for Clinical Use? JNCI. Journal of the National Cancer Institute.

[10] Bach, P. B., Kelley, M. J., Tate, R. C. & McCrory, D. C. Screening for lung cancer: a review of the current literature. Chest 123, 72S–82S, doi:S0012-3692(15)32984-6 (2003). 10.1378/chest.123.1_suppl.72s 12527566

[11] Silva, M., Pastorino, U. & Sverzellati, N. Lung cancer screening with low-dose CT in Europe: strength and weakness of diverse independent screening trials. Clin Radiol 72, 389–400, doi:S0009-9260(17)30015-610.1016/j.crad.2016.12.021 (2017). 10.1016/j.crad.2016.12.021 28168954

[12] Marcus PM (2015). Lung cancer screening with low dose computed tomography (LDCT): looking back and moving forward. Ann Transl Med.

[13] Schiller JH (2002). Comparison of four chemotherapy regimens for advanced non-small-cell lung cancer. N Engl J Med.

[14] Hirsch FR, Suda K, Wiens J, Bunn PA (2016). New and emerging targeted treatments in advanced non-small-cell lung cancer. Lancet.

[15] Fry AM (2002). The Nek2 protein kinase: a novel regulator of centrosome structure. Oncogene.

[16] Fry AM, O’Regan L, Sabir SR, Bayliss R (2012). Cell cycle regulation by the NEK family of protein kinases. J Cell Sci.

[17] Faragher AJ, Fry AM (2003). Nek2A kinase stimulates centrosome disjunction and is required for formation of bipolar mitotic spindles. Mol Biol Cell.

[18] Bahe S, Stierhof YD, Wilkinson CJ, Leiss F, Nigg EA (2005). Rootletin forms centriole-associated filaments and functions in centrosome cohesion. J Cell Biol.

[19] Xia, J. L., Machin, R. F., Gu, Z. M. & Zhan, F. H. Role of NEK2A in Human Cancer and Its Therapeutic Potentials. Biomed Res Int, doi:Artn 86246110.1155/2015/862461 (2015). 10.1155/2015/862461 PMC4330945 25705694

[20] Ning Z (2014). Abnormal expression of Nek2 in pancreatic ductal adenocarcinoma: a novel marker for prognosis. Int J Clin Exp Pathol.

[21] Zeng, Y. R. *et al*. Overexpression of NIMA-related kinase 2 is associated with progression and poor prognosis of prostate cancer. Bmc Urol 15, doi:ARTN 9010.1186/s12894-015-0085-7 (2015). 10.1186/s12894-015-0085-7 PMC4553013 26320076

[22] Lu L, Zhai X, Yuan R (2015). Clinical significance and prognostic value of Nek2 protein expression in colon cancer. Int J Clin Exp Pathol.

[23] He D, Xiang J, Li B, Liu H (2016). The dynamic behavior of Ect2 in response to DNA damage. Sci Rep.

[24] Luo Y (2015). Elevated expression of ECT2 predicts unfavorable prognosis in patients with colorectal cancer. Biomed Pharmacother.

[25] Santarella RA, Koffa MD, Tittmann P, Gross H, Hoenger A (2007). HURP wraps microtubule ends with an additional tubulin sheet that has a novel conformation of tubulin. J Mol Biol.

[26] Liao, W. J. *et al*. Silencing of DLGAP5 by siRNA Significantly Inhibits the Proliferation and Invasion of Hepatocellular Carcinoma Cells. PLoS One 8, doi:ARTN e80789 10.1371/journal.pone.0080789 (2013). 10.1371/journal.pone.0080789 PMC3851768 24324629

[27] Gomez, C. R. *et al*. Prognostic value of discs large homolog 7 transcript levels in prostate cancer. PLoS One 8, e82833, doi:10.1371/journal.pone.0082833 PONE-D-13-20821 (2013). 10.1371/journal.pone.0082833 PMC3857287 24349376

[28] Winn RA (2013). Reconstruction of an Integrated Genome-Scale Co-Expression Network Reveals Key Modules Involved in Lung Adenocarcinoma. PLoS One.

[29] Das TK (2013). Centrosomal kinase Nek2 cooperates with oncogenic pathways to promote metastasis. Oncogenesis.

[30] Justilien V (2017). Ect2-Dependent rRNA Synthesis Is Required for KRAS-TRP53-Driven Lung Adenocarcinoma. Cancer Cell.

[31] Zhong X (2014). Examining Nek2 as a better proliferation marker in non-small cell lung cancer prognosis. Tumor Biol.

[32] Albertson D (2008). Gene Expression Signature of Cigarette Smoking and Its Role in Lung Adenocarcinoma Development and Survival. PLoS One.

[33] Schneider, M. *et al*. AURKA, DLGAP5, TPX2, KIF11 and CKAP5: Five specific mitosis-associated genes correlate with poor prognosis for non-small cell lung cancer patients. International Journal of Oncology, doi:10.3892/ijo.2017.3834 (2017). 10.3892/ijo.2017.3834 PMC5238780 28101582

[34] Murata Y (2014). ECT2amplification and overexpression as a new prognostic biomarker for early-stage lung adenocarcinoma. Cancer Science.

[35] Hirata D (2009). Involvement of Epithelial Cell Transforming Sequence-2 Oncoantigen in Lung and Esophageal Cancer Progression. Clinical Cancer Research.

[36] Irizarry RA (2003). Summaries of Affymetrix GeneChip probe level data. Nucleic Acids Res.

[37] Gautier L, Cope L, Bolstad BM, Irizarry R (2004). A. affy - analysis of Affymetrix GeneChip data at the probe level. Bioinformatics.

[38] Gentleman, R. C. *et al*. Bioconductor: open software development for computational biology and bioinformatics. Genome Biol 5, R80, doi:gb-2004-5-10-r80 10.1186/gb-2004-5-10-r80 (2004). 10.1186/gb-2004-5-10-r80 PMC545600 15461798

[39] Yin JY (2016). Prediction models for platinum-based chemotherapy response and toxicity in advanced NSCLC patients. Cancer Lett.

