# Supplementary material for: Genome-scale analysis identifies NEK2, DLGAP5 and ECT2 as promising diagnostic and prognostic biomarkers in human lung cancer
Source: Sci Rep. 2017 Aug 14;7:8072. doi: 10.1038/s41598-017-08615-5 (PMC5556079; doi:10.1038/s41598-017-08615-5)
Supplement: Supplementary file 1 — Supplementary materials [file 41598_2017_8615_MOESM1_ESM.doc]

**Genome-scale analysis identifies NEK2, DLGAP5 and ECT2 as promising diagnostic and prognostic biomarkers in human lung cancer**

Yuan-Xiang Shi^1,2^, Ji-Ye Yin^1^, Yao Shen^1^, Wei Zhang^1^, Hong-Hao Zhou^1^, Zhao-Qian Liu^1,2*^

^1^Department of Clinical Pharmacology, Xiangya Hospital, Central South University, Changsha 410008; P. R. China;Institute of Clinical Pharmacology, Central South University, Hunan Key Laboratory of Pharmacogenetics, Changsha 410078; P. R. China; ^2^Hunan Province Cooperation Innovation Center for Molecular Target New Drug Study, Hengyang 421001, P.R.China.

*To whom correspondence should be addressed:

Professor Zhao-Qian Liu, Department of Clinical Pharmacology, Xiangya Hospital, Central South University, Changsha 410008; P. R. China; Institute of Clinical Pharmacology, Central South University; Hunan Key Laboratory of Pharmacogenetics, Changsha 410078; P. R. China. Tel: +86 731 84805380, Fax: +86 731 82354476, E-mail: [liuzhaoqian63@126.com](mailto:liuzhaoqian63@126.com).

**Supplementary materials：**

**
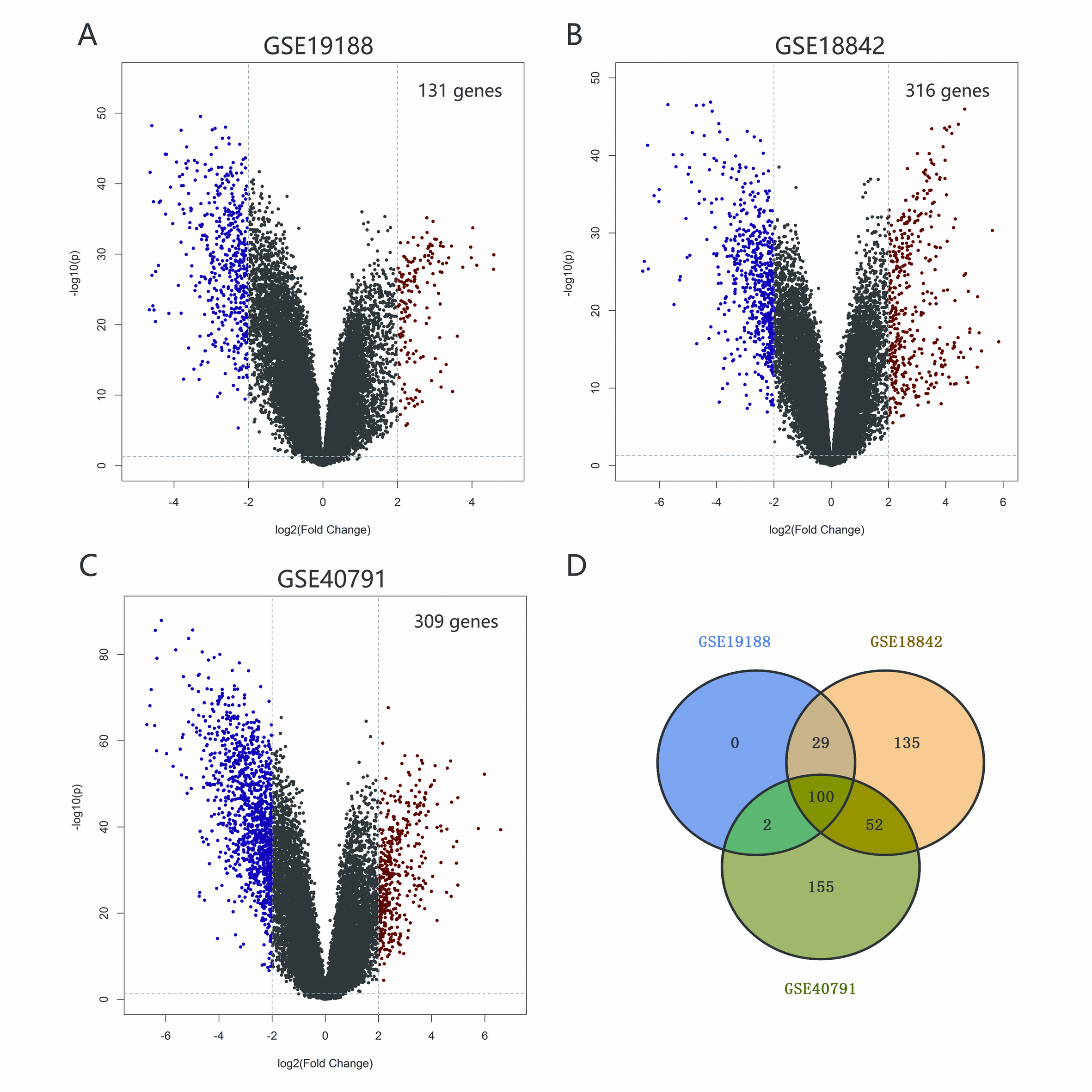
**

**Supplementary Figure1 Identification of expression differences between tumor and normal tissues. (A-C)** Volcano plot of the differential mRNA expression analysis. X-axis: log2 fold change; Y-axis: - log10 (FDR P-value) for each probes; Vertical dotted lines: fold change ≥ 4 or ≤ -4; Horizontal dotted line: the significance cutoff (FDR P-value = 0.05). **(A)** There were 131 genes up-regulated in GSE19188. **(B)** 316 genes up-regulated in GSE18842. **(C)** 309 genes up-regulated in GSE40791. **(D)** Overlap analysis between different datasets. There were 100 overlapping genes significantly differentially expressed between tumor and normal tissues in all three datasets.

**
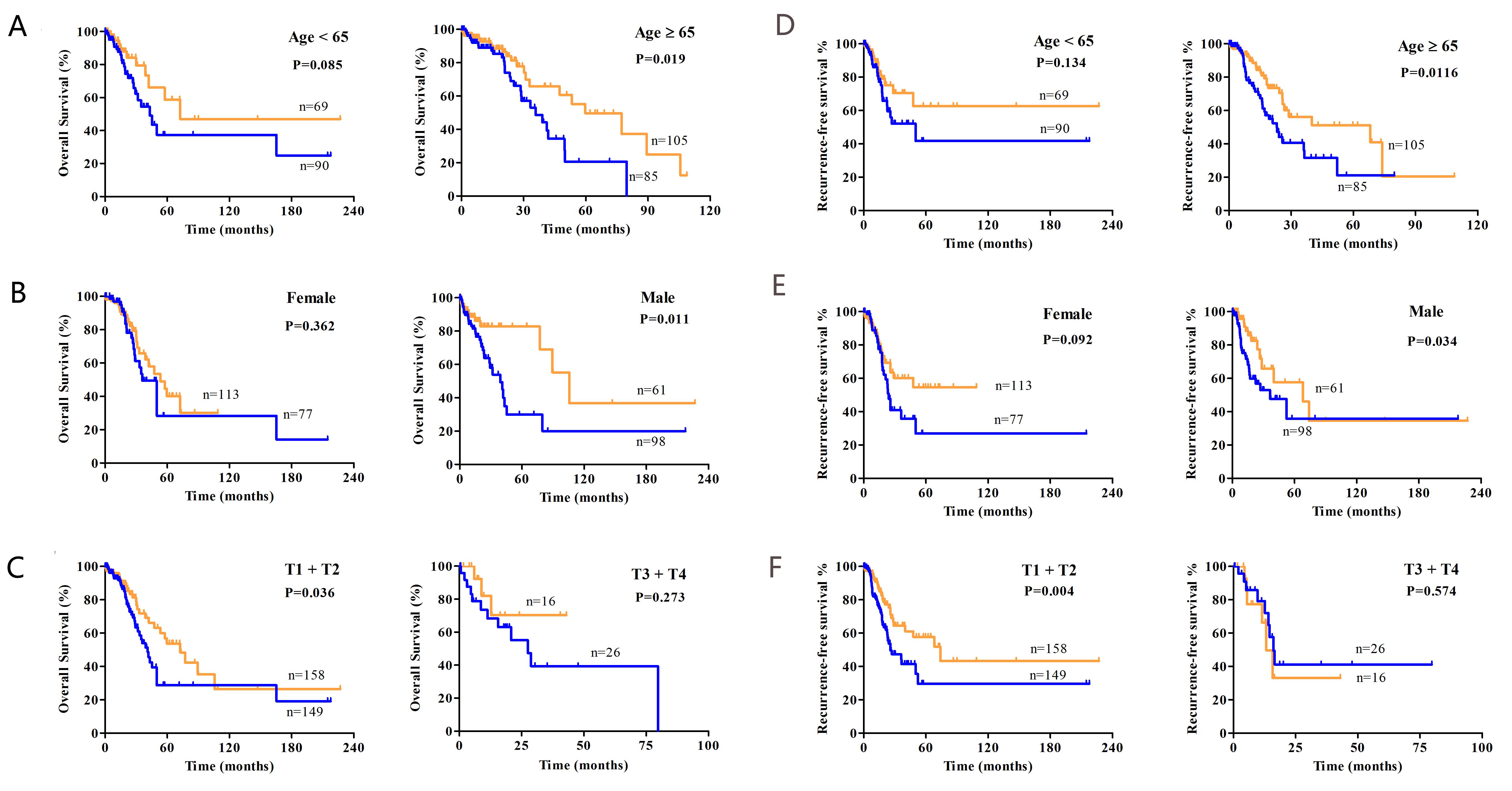
**

**Supplementary Figure2** Subgroup analysis in patients stratified by clinicopathological features was perfomed to explore the effect of NEK2 expression on OS and RFS. **(A)** Cumulative OS of patients in the age <65 group and in the age ≥65 group. (**B**) Cumulative OS of patients in female and in male. (**C**) Cumulative OS in T1+T2, T3+T4. **(D)** Cumulative RFS of patients in the age <65 group and in the age ≥65 group. (**E**) Cumulative RFS of patients in female and in male. (**F**) Cumulative RFS in T1+T2, T3+T4.

**
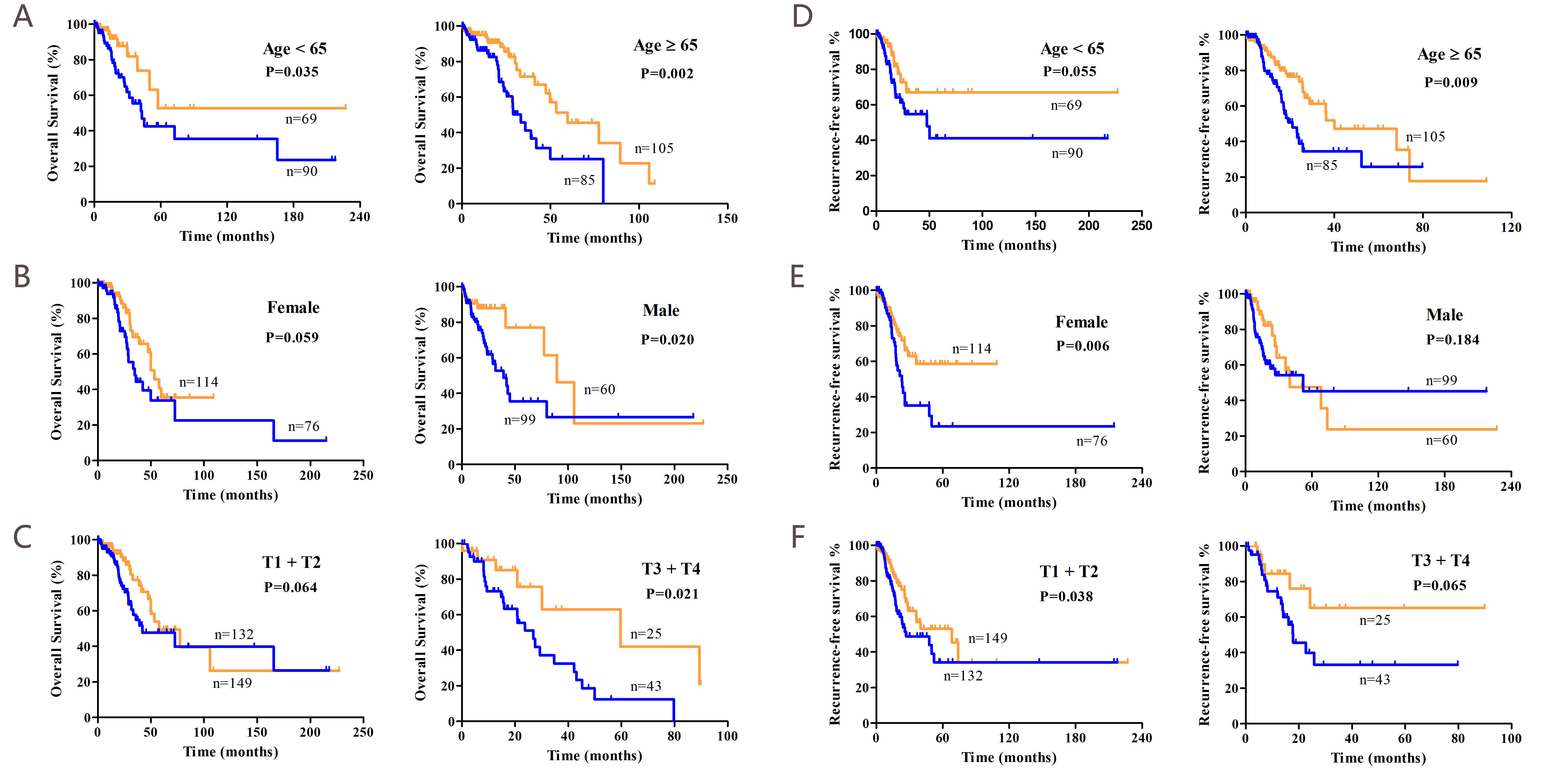
**

**Supplementary Figure3** Subgroup analysis in patients stratified by clinicopathological features was perfomed to explore the effect of DLGAP5 expression on OS and RFS. **(A)** Cumulative OS of patients in the age <65 group and in the age ≥65 group. (**B**) Cumulative OS of patients in female and in male. (**C**) Cumulative OS in T1+T2, T3+T4. **(D)** Cumulative RFS of patients in the age <65 group and in the age ≥65 group. (**E**) Cumulative RFS of patients in female and in male. (**F**) Cumulative RFS in T1+T2, T3+T4.

**
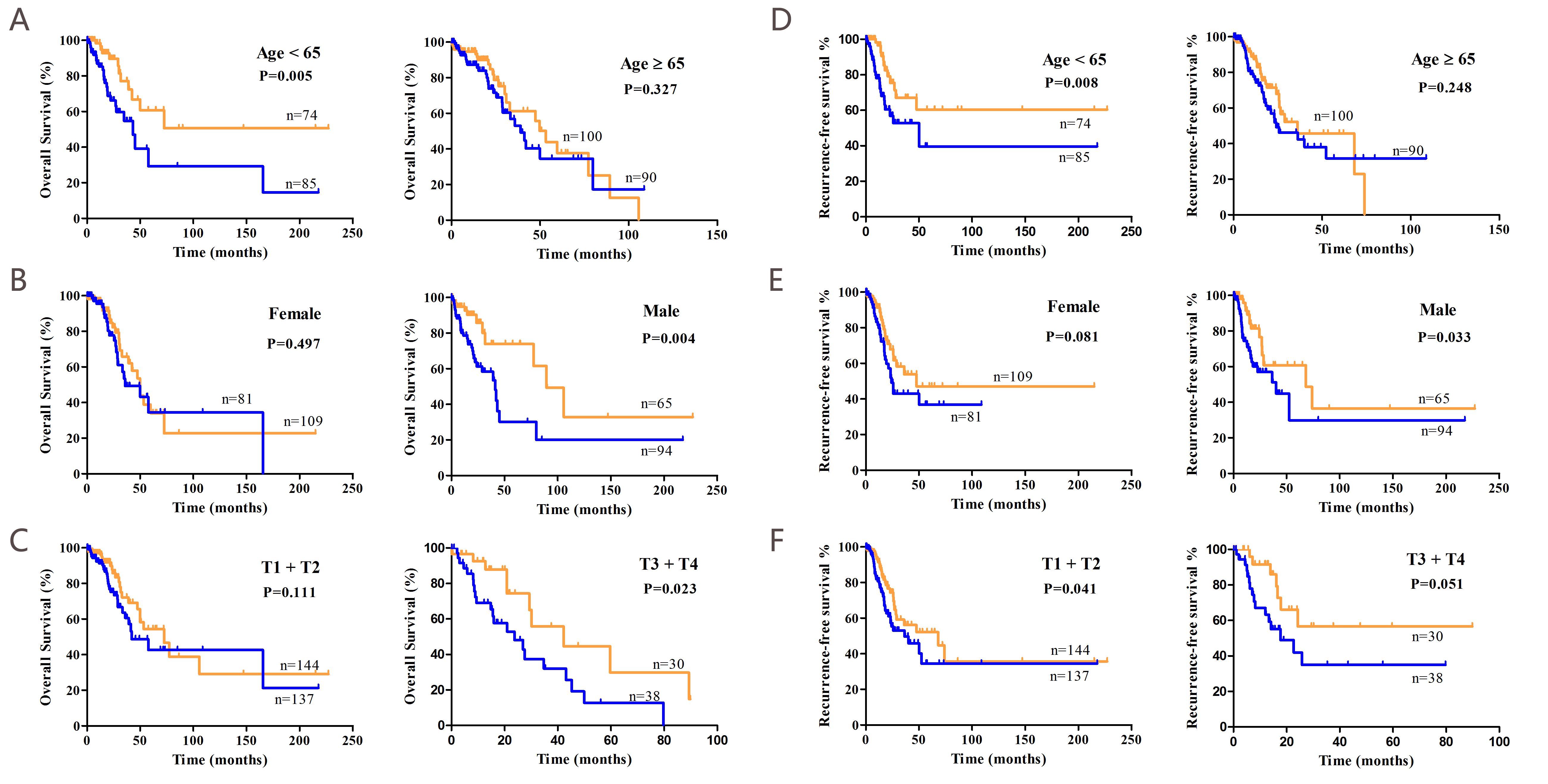
**

**Supplementary Figure4** Subgroup analysis in patients stratified by clinicopathological features was perfomed to explore the effect of ECT2 expression on OS and RFS. **(A)** Cumulative OS of patients in the age <65 group and in the age ≥65 group. (**B**) Cumulative OS of patients in female and in male. (**C**) Cumulative OS in T1+T2, T3+T4. **(D)** Cumulative RFS of patients in the age <65 group and in the age ≥65 group. (**E**) Cumulative RFS of patients in female and in male. (**F**) Cumulative RFS in T1+T2, T3+T4.

**Supplementary Table 1 Primers for qRT-PCR**

| **Gene** | **Primer** |
| --- | --- |
| **GAPDH** | F: CCCATCACCATCTTCCAGGAG |
|  | R: GTTGTCATGGATGACCTTGGC |
| **NEK2** | F: TGCTTCGTGAACTGAAACATCC |
|  | R: CCAGAGTCAACTGAGTCATCACT |
| **DLGAP5** | F: AAGTGGGTCGTTATAGACCTGA |
|  | R: TGCTCGAACATCACTCTCGTTAT |
| **ECT2** | F: ACTACTGGGAGGACTAGCTTG |
|  | R: CACTCTTGTTTCAATCTGAGGCA |

F: Forward primer, R: Reverse primer

**Supplementary Table 2 100-gene-list.**

| Gene.symbol | Gene.title |
| --- | --- |
| ADAM12 | ADAM metallopeptidase domain 12 |
| ADAMDEC1 | ADAM-like, decysin 1 |
| AK4 | Adenylate kinase 4 |
| AKR1B10 | Aldo-keto reductase family 1, member B10 (aldose reductase) |
| ANLN | Anillin, actin binding protein |
| ASPM | Asp (abnormal spindle) homolog, microcephaly associated (Drosophila) |
| ATAD2 | ATPase family, AAA domain containing 2 |
| AURKA | Aurora kinase A |
| BIRC5 | Baculoviral IAP repeat containing 5 |
| BUB1 | BUB1 mitotic checkpoint serine/threonine kinase |
| BUB1B | BUB1 mitotic checkpoint serine/threonine kinase B |
| CCNA2 | Cyclin A2 |
| CCNB1 | Cyclin B1 |
| CCNB2 | Cyclin B2 |
| CDC20 | Cell division cycle 20 |
| CDCA3 | Cell division cycle associated 3 |
| CDCA5 | Cell division cycle associated 5 |
| CDCA7 | Cell division cycle associated 7 |
| CDK1 | Cyclin-dependent kinase 1 |
| CDKN3 | Cyclin-dependent kinase inhibitor 3 |
| CENPA | Centromere protein A |
| CENPF | Centromere protein F, 350/400kDa |
| CENPU | Centromere protein U |
| CEP55 | Centrosomal protein 55kDa |
| CHEK1 | Checkpoint kinase 1 |
| COCH | Cochlin |
| COL10A1 | Collagen, type X, alpha 1 |
| COL11A1 | Collagen, type XI, alpha 1 |
| COL1A1 | Collagen, type I, alpha 1 |
| CTHRC1 | Collagen triple helix repeat containing 1 |
| CXCL13 | Chemokine (C-X-C motif) ligand 13 |
| DEPDC1 | DEP domain containing 1 |
| DLGAP5 | Discs, large (Drosophila) homolog-associated protein 5 |
| DSP | Desmoplakin |
| ECT2 | Epithelial cell transforming 2 |
| EGLN3 | Egl-9 family hypoxia-inducible factor 3 |
| EZH2 | Enhancer of zeste 2 polycomb repressive complex 2 subunit |
| FAM72 | Family with sequence similarity 72 |
| FANCI | Fanconi anemia, complementation group I |
| FERMT1 | Fermitin family member 1 |
| GINS1 | GINS complex subunit 1 (Psf1 homolog) |
| GINS2 | GINS complex subunit 2 (Psf2 homolog) |
| GJB2 | Gap junction protein, beta 2, 26kDa |
| GPR87 | G protein-coupled receptor 87 |
| GPX2 | Glutathione peroxidase 2 (gastrointestinal) |
| GREM1 | Gremlin 1, DAN family BMP antagonist |
| GTSE1 | G-2 and S-phase expressed 1 |
| HJURP | Holliday junction recognition protein |
| HMGB3 | High mobility group box 3 |
| HMMR | Hyaluronan-mediated motility receptor (RHAMM) |
| HS6ST2 | Heparan sulfate 6-O-sulfotransferase 2 |
| IGF2BP3 | Insulin-like growth factor 2 mRNA binding protein 3 |
| KIAA0101 | KIAA0101 |
| KIF11 | Kinesin family member 11 |
| KIF14 | Kinesin family member 14 |
| KIF20A | Kinesin family member 20A |
| KIF23 | Kinesin family member 23 |
| KIF2C | Kinesin family member 2C |
| KIF4A | Kinesin family member 4A |
| KRT6A | Keratin 6A |
| LINC00673 | Long intergenic non-protein coding RNA 673 |
| MAD2L1 | MAD2 mitotic arrest deficient-like 1 (yeast) |
| MELK | Maternal embryonic leucine zipper kinase |
| MKI67 | Marker of proliferation Ki-67 |
| MMP1 | Matrix metallopeptidase 1 (interstitial collagenase) |
| MMP12 | Matrix metallopeptidase 12 (macrophage elastase) |
| NCAPG | Non-SMC condensin I complex, subunit G |
| NDC80 | NDC80 kinetochore complex component |
| NEK2 | NIMA-related kinase 2 |
| NMU | Neuromedin U |
| NUF2 | NUF2, NDC80 kinetochore complex component |
| NUSAP1 | Nucleolar and spindle associated protein 1 |
| PBK | PDZ binding kinase |
| PRC1 | Protein regulator of cytokinesis 1 |
| PSAT1 | Phosphoserine aminotransferase 1 |
| PTTG1 | Pituitary tumor-transforming 1 |
| RAD51AP1 | RAD51 associated protein 1 |
| RMI2 | RecQ mediated genome instability 2 |
| RRM2 | Ribonucleotide reductase M2 |
| S100A2 | S100 calcium binding protein A2 |
| SCG5 | Secretogranin V (7B2 protein) |
| SIX1 | SIX homeobox 1 |
| SLC2A1 | Solute carrier family 2 (facilitated glucose transporter), member 1 |
| SPP1 | Secreted phosphoprotein 1 |
| SRD5A1 | Steroid-5-alpha-reductase, alpha polypeptide 1 |
| SULF1 | Sulfatase 1 |
| TFAP2A | Transcription factor AP-2 alpha (activating enhancer binding protein 2 alpha) |
| TMPRSS4 | Transmembrane protease, serine 4 |
| TOP2A | Topoisomerase (DNA) II alpha 170kDa |
| TPX2 | TPX2, microtubule-associated |
| TRIP13 | Thyroid hormone receptor interactor 13 |
| TTK | TTK protein kinase |
| TYMS | Thymidylate synthetase |
| UBE2C | Ubiquitin-conjugating enzyme E2C |
| UBE2T | Ubiquitin-conjugating enzyme E2T (putative) |
| UCHL1 | Ubiquitin carboxyl-terminal esterase L1 (ubiquitin thiolesterase) |
| UGT8 | UDP glycosyltransferase 8 |
| UHRF1 | Ubiquitin-like with PHD and ring finger domains 1 |
| WDR72 | WD repeat domain 72 |
| ZWINT | ZW10 interacting kinetochore protein |

**Supplementary Table 3 Logistic regression analysis of ROC analyses in TCGA dataset.**

| **Variable** | **HR** | **P-value** |
| --- | --- | --- |
| NEK2 (low vs. high) | 7.567 | **< 0.001** |
| Age (<65 vs. ≥65 ) | 0.568 | 0.336 |
| Gender (Female vs. Male) | 0.603 | 0.351 |
| Clinical stage (I–II vs. III–IV) | 0.391 | 0.183 |
| Smoking history (yes vs. no) | 1.314 | 0.622 |
|  |  |  |
| DLGAP5 (low vs. high) | 7.088 | **< 0.001** |
| Age (<65 vs. ≥65 ) | 0.669 | 0.480 |
| Gender (Female vs. Male) | 0.799 | 0.694 |
| Clinical stage (I–II vs. III–IV) | 0.528 | 0.335 |
| Smoking history (yes vs. no) | 1.603 | 0.406 |
|  |  |  |
| ECT2 (low vs. high) | 38.445 | **< 0.001** |
| Age (<65 vs. ≥65 ) | 0.877 | 0.788 |
| Gender (Female vs. Male) | 0.498 | 0.178 |
| Clinical stage (I–II vs. III–IV) | 0.566 | 0.361 |
| Smoking history (yes vs. no) | 2.751 | 0.052 |
